# Longitudinal Association Between Perceived Fatigability and Brain Volumes in Community-Dwelling Older Adults

**DOI:** 10.1093/geroni/igab046.800

**Published:** 2021-12-17

**Authors:** Jennifer Schrack, Fangyu Liu, Yang An, Amal Wanigatunga, Alden Gross, Eleanor Simonsick, Luigi Ferrucci, Susan Resnick

**Affiliations:** 1 Johns Hopkins Bloomberg School of Public Health, Baltimore, Maryland, United States; 2 Johns Hopkins University, Baltimore, Maryland, United States; 3 National Institute on Aging, Baltimore, Maryland, United States; 4 National Instute on Aging/NIH, Baltimore, Maryland, United States

## Abstract

Perceived fatigability is linked to declining physical and cognitive performance, yet whether fatigability reflects early subclinical change in brain structure is unknown. Using mixed effects models, we assessed the longitudinal association of 3T MRI-derived brain volumes with perceived fatigability after a 5-min treadmill walk (0.67 m/s, 0% grade) using the Borg Rating of Perceived Exertion scale (range 6-20) in 802 BLSA participants (age 68.2+/-12.4 years, 45% men 66% White). In models adjusted for intracranial volume, demographics, chronic conditions, and CESD score, declining gray matter volumes in the frontal (β=-0.01) and temporal (β=-0.02) lobes, as well as the hippocampus (β=-0.25), precuneus (β=-0.10) and thalamus (β=-0.19) were associated with higher fatigability. Larger ventricular volumes were also associated with higher fatigability (β=0.02). Brain atrophy, particularly in gray matter and the hippocampal region, is longitudinally associated with increased fatigability in cognitively normal older adults, making it a potential marker of brain atrophy.

